# LncRNA TINCR attenuates cardiac hypertrophy by epigenetically silencing CaMKII

**DOI:** 10.18632/oncotarget.17735

**Published:** 2017-05-10

**Authors:** Mingjing Shao, Guangdong Chen, Fengli Lv, Yanyan Liu, Hongjun Tian, Ran Tao, Ronghuan Jiang, Wei Zhang, Chuanjun Zhuo

**Affiliations:** ^1^ National Integrated Traditional and Western Medicine Center for Cardivascular Disease, China-Japan Friendship Hospital, Beijing, China; ^2^ Department of Psychological Medicine, Wenzhou Seventh People's Hospital, Wenzhou, China; ^3^ Department of Rehabilitation, The First Affiliated Hospital of Tianjin University of Traditional Chinese Medicine, Tianjin, China; ^4^ Department of Psychological Medicine, Tianjin Anning Hospital, Tianjin, China; ^5^ Department of Psychological Medicine, Tianjin Anding Hospital, Tianjin, China; ^6^ Department of Psychological Medicine, Beijing Shijian Integrated Medicine Science Institute, Beijing, China; ^7^ Department of Psychological Medicine, Chinese People's Liberation Army General Hospital, Beijing, China; ^8^ Department of Psychological Medicine, Chinese Land Force General Hospital, Beijing, China; ^9^ Department of Psychological Medicine, Chinese People's Liberation Army, Medical School, Beijing, China

**Keywords:** TINCR, CaMKII, EZH2, cardiac hypertrophy

## Abstract

In the previous study, we established a mouse model of cardiac hypertrophy using transverse aortic constriction (TAC) and found that the expression of long non-coding RNAs TINCR was downregulated in myocardial tissue. The present study was designed to determine the potential role of TINCR in the pathogenesis of cardiac hypertrophy. Our results showed that enforced expression of TINCR could attenuate cardiac hypertrophy in TAC mice. Angiotensin II (Ang-II) was found to be associated with reduced TINCR expression and increased hypertrophy in cultured neonatal cardiomyocytes. RNA-binding protein immunoprecipitation assay confirmed that TINCR could directly bind with EZH2 in cardiomyocytes. The results of chromatin immunoprecipitation assay revealed that EZH2 could directly bind to CaMKII promoter region and mediate H3K27me3 modification. Knockdown of TINCR was found to reduce EZH2 occupancy and H3K27me3 binding in the promoter of CaMKII in cardiomyocytes. In addition, enforced expression of TINCR was found to decrease CaMKII expression and attenuate Ang-II-induced cardiomyocyte hypertrophy. Furthermore, our results also showed that Ang-II could increase CaMKII expression in cardiomyocytes, which consequently contributed to cellular hypertrophy. In conclusion, our findings demonstrated that TINCR could attenuate myocardial hypertrophy by epigenetically silencing of CaMKII, which may provide a novel therapeutic strategy for cardiac hypertrophy.

## INTRODUCTION

Myocardial hypertrophy is an adaptive reaction in response to increased afterload of the heart to maintain cardiac systolic function at an early stage. However, sustained myocardial hypertrophy is associated with maladaptive ventricular remodelling and increased incidence of heart failure. In recent years, although a number of peptide hormones and growth factors have been identified to be involved in the modulation of cardiac hypertrophy [1], the potential molecular mechanisms of myocardial hypertrophy are not fully elucidated.

Long non-coding RNAs (lncRNAs) are transcribed RNA molecules > 200 nucleotides in length without known protein-coding function. They can regulate the expression of target genes at epigenetic, transcriptional, and post-transcriptional levels [2]. Previous studies have reported that lncRNAs play critical roles in the modulation of heart development and cardiovascular diseases. However, only a limited number of lncRNAs have been found to be associated with myocardial hypertrophy. Wang et al. indicated that lncRNA CHRF was involved in the regulation of cardiac hypertrophy by targeting miR-489 [3]. In addition, Han et al. found that lncRNA Mhrt could protect the heart against pathological hypertrophy [4].

In the previous study, we generated a mouse model of cardiac hypertrophy using transverse aortic constriction (TAC) and conducted a microarray to identify the differentially expressed lncRNAs in myocardial tissue. Our results showed that terminal differentiation-induced ncRNA (TINCR) was remarkably downregulated in hypertrophic mice. The TINCR gene locates on chromosome 19 in humans and encodes a 3.7-kb lncRNA. It has been reported that TINCR-deficient epidermis lacks terminal differentiation ultrastructure, such as keratohyalin granules and intact lamellar bodies [5]. The present study was aimed to determine the potential role of TINCR in the pathogenesis of cardiac hypertrophy.

## RESULTS

**Figure 1:**
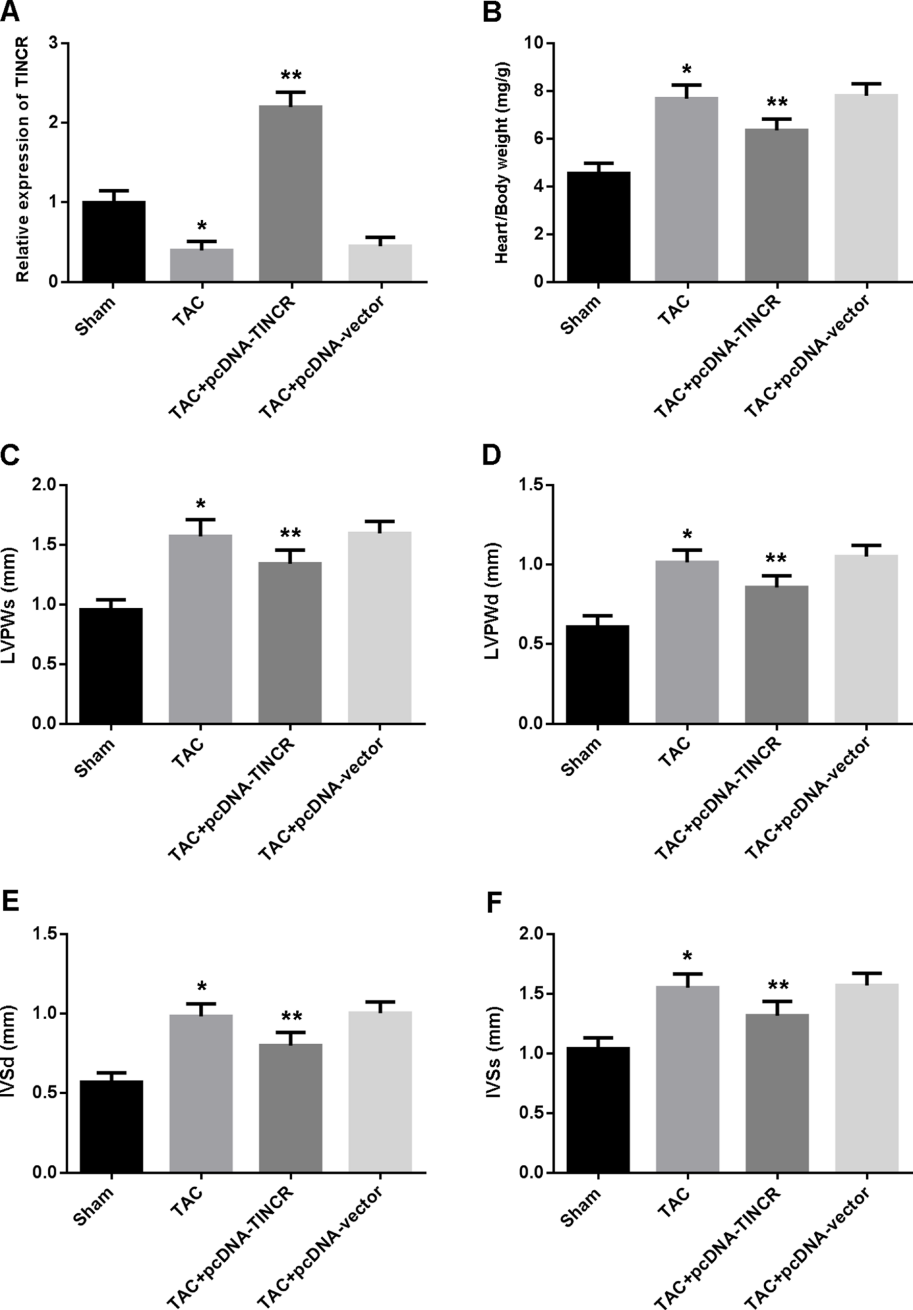
(**A**) The expression of TINCR in myocardium determined by real-time PCR; (**B**) The ratios of heart weight to body weight; (**C**–**F**) Echocardiographic evaluation of cardiac hypertrophy. LVPWs = left ventricular posterior wall thickness at end-systole; LVPWd = left ventricular posterior wall thickness at end-diastole; IVSd = interventricular septum thickness at end-diastole; IVSs = interventricular septum thickness at end-systole. *P < 0.05, vs. Sham; ^*^P < 0.05, vs. TAC. (*n* = 5).

The expression of TINCR was reduced in the myocardium of hypertrophic mice and increased after transfection with lentivirus pcDNA-TINCR (Figure 1A). Cardiac hypertrophy was evaluated by echocardiography and the ratio of heart weight to body weight was measured. The results showed that heart/body ratio, LVPWd, LVPWs, IVSd and IVSs were significantly increased in the TAC group compared to the control group, while TINCR overexpression was found to attenuate cardiac hypertrophy in the TAC mice (Figure 1B–1F).

**Figure 2:**
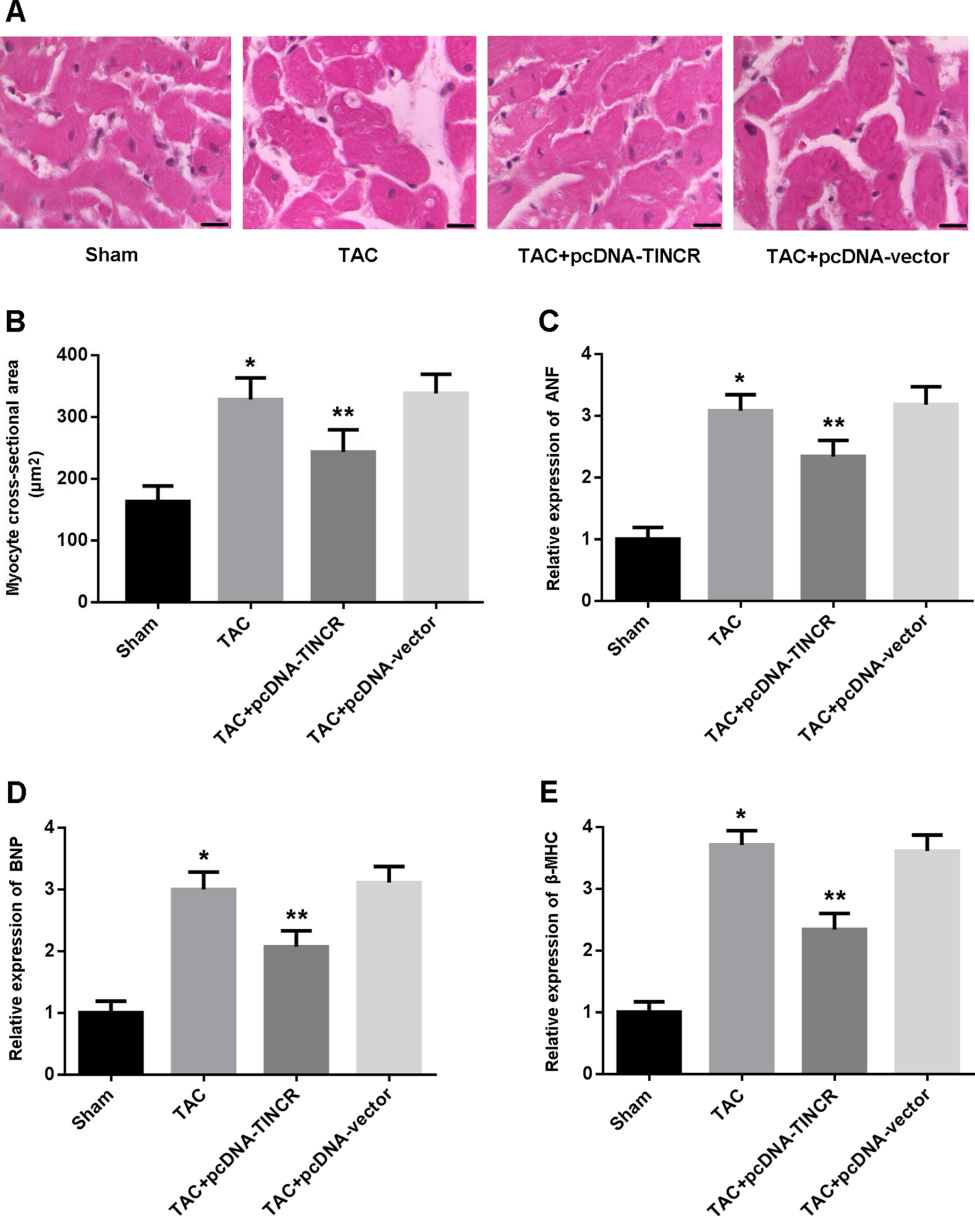
(**A**) Representative images of left ventricular tissue stained with hematoxylin-eosin (scale bar: 10 μm); (**B**) Quantitative analysis of myocyte cross-sectional area; (**C**–**E**) Relative expression of ANF, BNP and β-MHC detected by real-time PCR. * *P* < 0.05, vs. Sham; ^*^P < 0.05, vs. TAC (*n* = 5).

Left ventricular tissue was stained with hematoxylin/eosin and myocyte cross-sectional area (CSA) was calculated. The results indicated that CSA was markedly increased in the TAC mice and reduced following treatment with lentivirus pcDNA-TINCR (Figure 2A, 2B). ANF, BNP and β-MHC, as indicators of myocardial hypertrophy, were found to be upregulated in the TAC group and downregulated in the TAC+pcDNA-TINCR group (Figure 2C, 2D).

**Figure 3:**
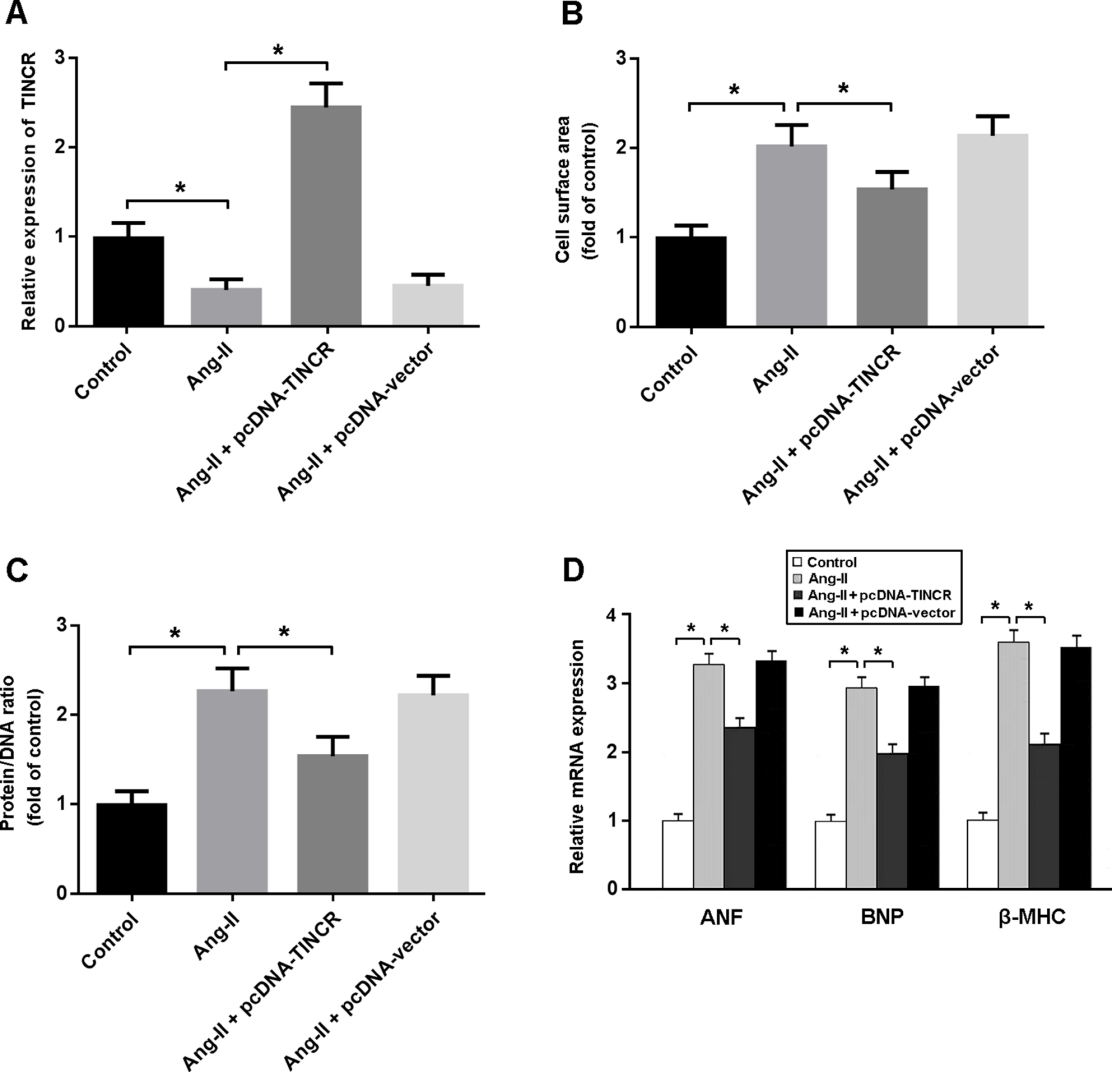
(**A**) Cardiomyocytes were transfected with adenoviral pcDNA-TINCR or empty vector and then treated with Ang-II at 150 nM for 24 h. The TINCR expression was determined by real-time PCR. (**B**, **C**) Cell surface area and protein/DNA ratio were measured to evaluate cardiomyocyte hypertrophy; (**D**) Relative mRNA expression of ANF, BNP and β-MHC in cardiomyocytes. * *P* < 0.05.

In this study, angiotensin II (Ang-II) was used to induce cardiomyocyte hypertrophy. As shown in Figure 3, there were decreased expression of TINCR and increased cell surface area, protein/DNA ratio and hypertrophy-associated gene expression in cardiomyocytes treated with Ang-II. However, enforced expression of TINCR was found to attenuate Ang-II-induced cardiomyocyte hypertrophy.

**Figure 4:**
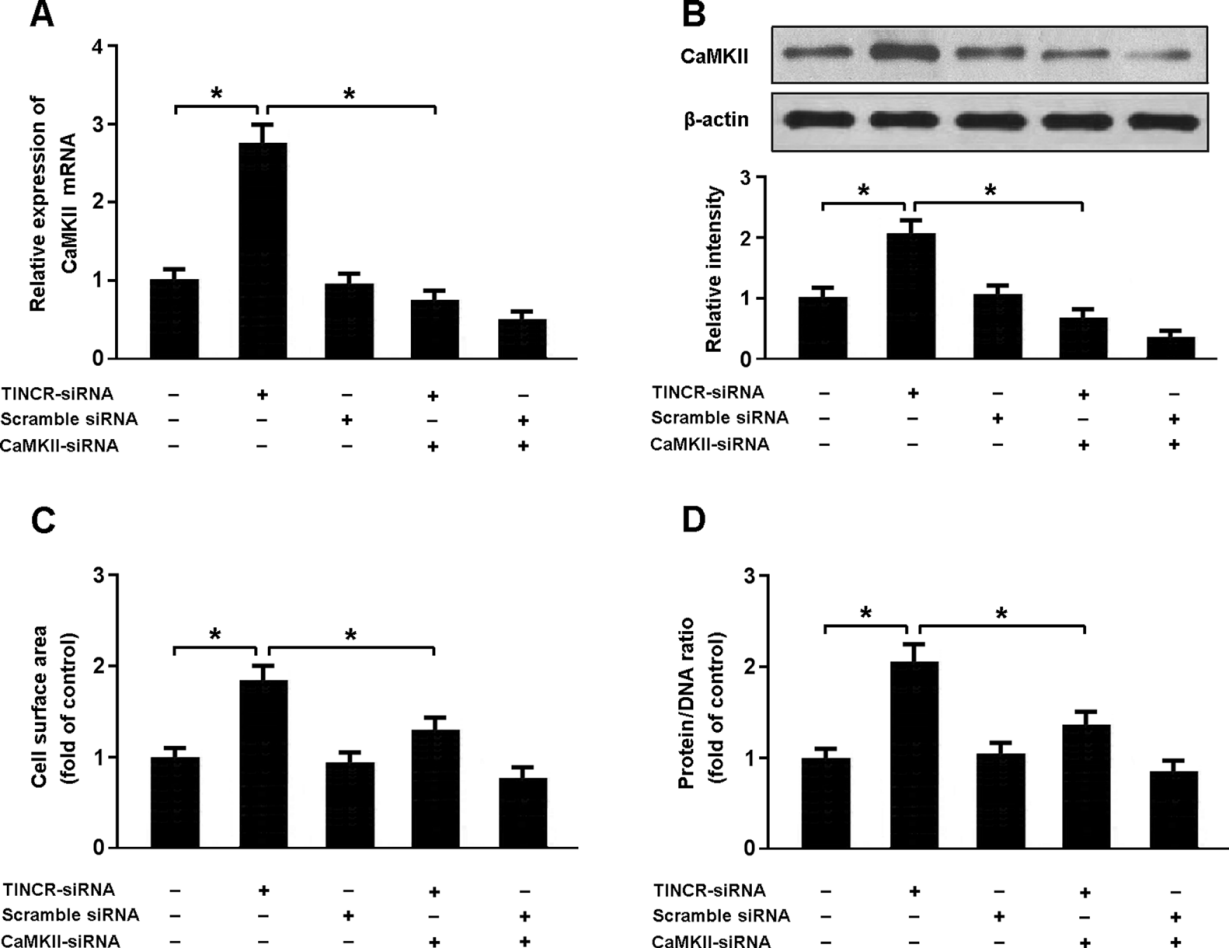
(**A**, **B**) Cardiomyocytes were infected with adenoviral TINCR-siRNA or scramble siRNA and then transfected with adenoviral CaMKII-siRNA. The expression of CaMKII was detected by real-time PCR and Western blot. (**C**, **D**) Cell surface area and protein/DNA ratio were measured to access cardiomyocyte hypertrophy. *P < 0.05.

As shown in Figure 4, the expression of CaMKII was increased in cardiomyocytes transfected with TINCR-siRNA, consequently leading to increased cellular hypertrophy. However, knockdown of CaMKII was found to inhibit hypertrophy in cardiomyocytes with TINCR-siRNA transfection, which suggests that TINCR downregulation could induce cardiomyocyte hypertrophy by upregulating the expression of CaMKII.

**Figure 5:**
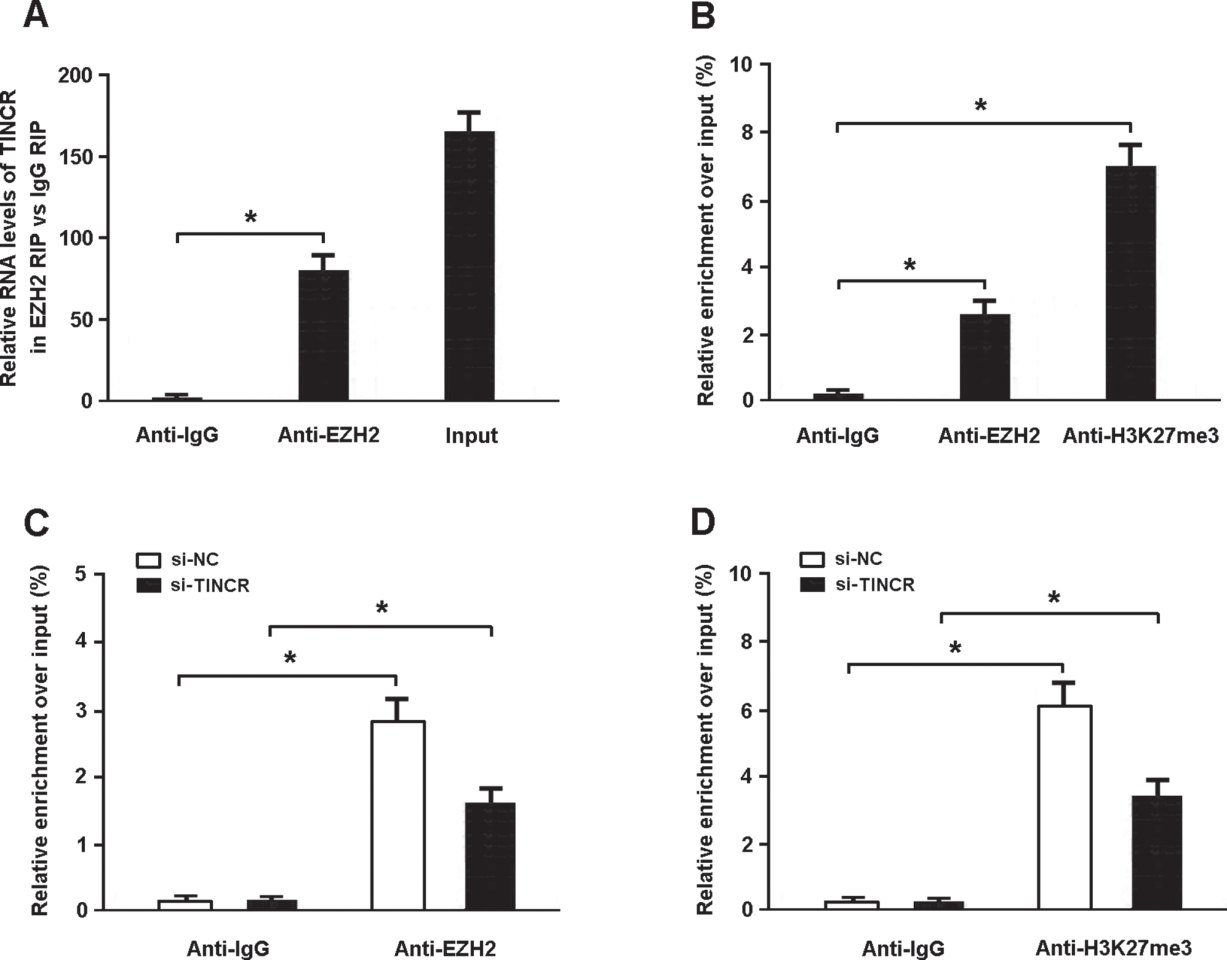
(**A**) The RIP assay was conducted to confirm whether TINCR could directly bind with EZH2. (**B**) The ChIP assay was performed to verify whether EZH2 could directly bind to CaMKII promoter and mediate H3K27me3 modification. (**C**, **D**) ChIP-qPCR of EZH2 occupancy and H3K27me3 binding in the promoter of CaMKII in cardiomyocytes transfected with TINCR siRNA or scrambled siRNA. *P < 0.05.

To confirm whether TINCR suppresses the expression of CaMKII through binding to PRC2, we carried out RIP assay and found that TINCR could directly bind to EZH2 in myocardial cells (Figure 5A). The results of ChIP assay indicated that EZH2 could directly bind to CaMKII promoter and induce H3K27me3 modification (Figure 5B). In addition, TINCR knockdown was found to inhibit EZH2 occupancy and H3K27me3 binding in the CaMKII promoter region (Figure 5C, 5D).

**Figure 6:**
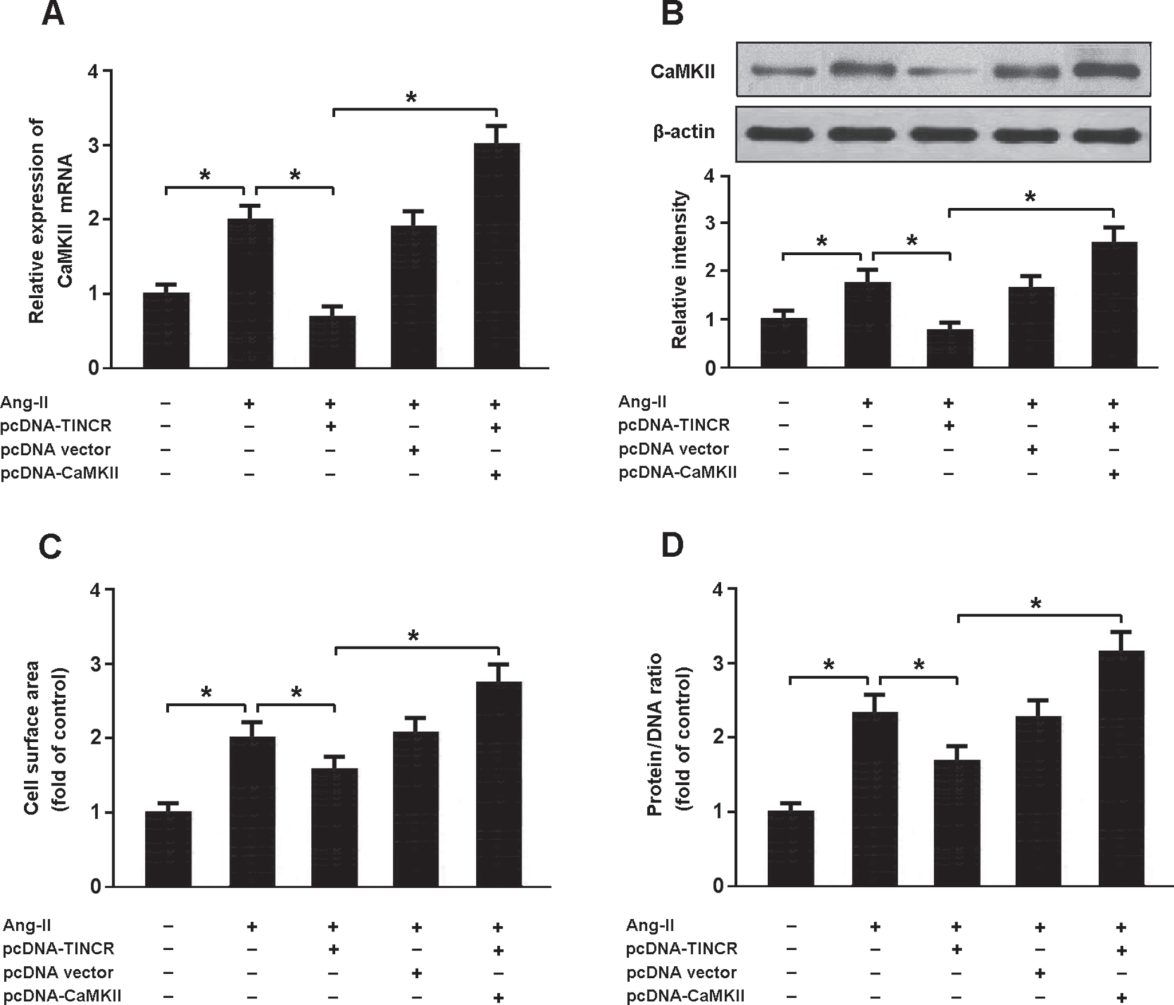
(**A**, **B**) Cardiomyocytes were transfected with pcDNA-TINCR and/or pcDNA-CaMKII prior to treatment with Ang-II and the expression of CaMKII was analyzed using real-time PCR and Western blot. (**C**, **D**) Cell surface area and protein/DNA ratio were measured to evaluate cardiomyocyte hypertrophy. *P < 0.05.

Myocardial cells were transfected with pcDNA-TINCR and pcDNA-CaMKII, and then treated with Ang-II. The findings showed that Ang-II could increase CaMKII expression and induce cardiomyocyte hypertrophy. Enforced expression of TINCR was found to reduce CaMKII expression and inhibit hypertrophy in cardiomyocytes treated with Ang-II. In addition, overexpression of TINCR and CaMKII could promote cardiomyocyte hypertrophy, suggesting that Ang-II might induce cardiomyocyte hypertrophy by regulating TINCR/CaMKII pathway (Figure 6A–6D).

## DISCUSSION

In recent years, a variety of lncRNAs have been identified to be involved in the regulation of cardiovascular diseases [6–8], but it remains unclear how lncRNAs function in the development of cardiac hypertrophy. In this study, we generated a mouse model of cardiac hypertrophy using TAC method and found that lncRNA TINCR was downregulated in the myocardial tissue. We then carried out *in vivo* and *in vitro* experiments to investigate the molecular mechanisms by which TINCR is involved in the regulation of myocardial hypertrophy. Our results indicated that TINCR could inhibit cardiac hypertrophy by epigenetically silencing of CaMKII.

TINCR, a lncRNA isolated from human well differentiated somatic tissues, controls epidermal differentiation and is downregulated in squamous cell carcinoma [5, 9]. Recently, Xu et al. reported that TINCR could bind to staufen1 protein and affect KLF2 mRNA stability, then KLF2 regulated the transcription of cyclin-dependent kinase genes P21 and P15, thereby modulating the proliferation and apoptosis of gastric carcinoma cells [10]. In addition, Zhang et al. revealed that downregulation of TINCR promoted proliferation and metastasis in colorectal cancer and TINCR could be considered as a potential cancer suppressor gene [11]. Furthermore, Chen et al. demonstrated that TINCR could promote bladder cancer development and progression and TINCR knockdown might provide a novel therapeutic strategy for bladder cancer [12].

There is growing evidence that PRC2 participates in various biological processes, such as differentiation, proliferation and apoptosis [13]. EZH2 has been reported to be a key catalytic subunit of PRC2 and function as a histone methyltransferase inducing histone H3 lysine 27 trimethylation (H3K27me3) [14]. As an important epigenetic regulator, EZH2 is widely expressed in a number of human cancers and regulates the expression of target genes involved in proliferation, differentiation, and neoplastic cell transformation [15]. In the present study, our findings revealed that TINCR could epigenetically inhibit the transcription of CaMKII by binding to PRC2 and recruiting it to the promoter of CaMKII.

CaMKII, a key serine/threonine protein kinase mainly expressed in the heart, is critically involved in the regulation of ion channels, transcription factors and other membrane proteins which are crucial for myocardial structure and electrical activity [16]. It has been well documented that CaMKII can function as an inducer of cardiac hypertrophy. In previous studies, CaMKII transgenic mice showed significant myocardial hypertrophy, and deletion of CaMKII was found to attenuate pathological hypertrophy [17, 18]. Moreover, Toko et al. revealed that CaMKII played an important role in the progression of heart failure by accumulation of p53 and promotion of cellular apoptosis in dilated cardiomyopathy [19]. In the present study, our findings suggested that CaMKII was upregulated in hypertrophic myocardium, and it might be epigenetically regulated by TINCR and involved in the pathogenesis of myocardial hypertrophy.

In conclusion, our study revealed that CaMKII is a new target of suppression by EZH2-mediated H3K27me3 and is epigenetically inhibited by TINCR, which will provide a novel therapeutic strategy for cardiac hypertrophy.

## MATERIALS AND METHODS

### Animal model and treatment

All experiments were approved by the Animal Ethics Committee and were performed in accordance with the Guide for the Care and Use of Laboratory Animals. TAC surgeries were carried out on 8 weeks old male C57BL/6 N mice. After thoracotomy, the transverse thoracic aorta was dissected, and a 6–0 silk suture was tied around the aorta against a 26-gauge needle. The sham groups underwent a sham operation involving thoracotomy and aortic dissection without constriction of the aorta. The mice were then divided into 4 groups: Sham group, TAC group, TAC+pcDNA-TINCR group (TAC mice were intracoronary administered with 80 μL lentivirus pcDNA-TINCR) and TAC+ pcDNA-vector group (TAC mice were treated with 80 μL lentivirus pcDNA vector). After 4 weeks, echocardiography was conducted and the hearts were harvested for analysis.

### Cardiomyocyte culture

Neonatal ventricular myocytes were isolated as previously described [20]. Briefly, myocardial tissues were surgically removed and dispersed in a series of incubations. After centrifugation, the cells were suspended in Dulbecco's modified Eagle medium/F-12. The dissociated cells were pre-plated at 37°C for 1 h to separate cardiomyocytes by adherence of cardiac fibroblasts. The cardiomyocytes were collected and diluted to 1 × 10^6^ cells/mL and plated in 1% gelatin-coated different culture dishes.

### Echocardiographic study

Echocardiography was conducted in M-mode using an echocardiography system with a 15 MHz linear transducer (Vevo 2100; VisualSonics, Canada). Cardiac hypertrophy was evaluated by measurement of heart size, including left ventricular posterior wall thickness at end-systole (LVPWs), left ventricular posterior wall thickness at end-diastole (LVPWd), interventricular septum thickness at end-diastole (IVSd) and interventricular septum thickness at end-systole (IVSs). All measurements were averaged for 3 consecutive cardiac cycles.

### Histological analysis

Left ventricular tissue was surgically removed, fixed in 10% buffered formalin, embedded in paraffin, and sliced into 5-μm-thick sections. Slides were then stained with hematoxylin-eosin and observed under a light microscope.

### Chromatin immunoprecipitation (ChIP)

The ChIP assay was performed using the EZ-ChIP Kit (Millipore, USA). Briefly, cardiomyocytes were treated with formaldehyde and incubated for 10 minutes to generate DNA-protein cross-links. Cell lysates were sonicated to generate chromatin fragments of 200–300 bp and immunoprecipitated with EZH2 and H3K27me3 -specific antibodies (Millipore, USA). The precipitated chromatin DNA was purified and subjected to quantitative PCR analysis for enrichment of the target sequences.

### RNA-binding protein immunoprecipitation (RIP)

Cardiomyocytes were lysed in RIP lysis buffer, following incubation with RIP buffer consisting of 150 mM KCl, 25 mM Tris pH 7.4, 5 mM EDTA, 0.5 mM DTT, 0.5% NP40, 100 U/ml RNAase inhibitor SUPERASin. The RIP buffer contained magnetic beads conjugated with anti-EZH2 antibody (Millipore, USA) or negative control IgG. The samples were incubated with Proteinase K and then immunoprecipitated RNA was isolated. Purified RNA was subjected to real-time PCR to determine the presence of binding targets using respective primers.

### Real-time PCR

Total RNA was isolated from myocardial tissue using TRIzol reagent (Invitrogen, USA). RNA was reverse transcribed using SuperScript First-Strand cDNA System (Invitrogen, USA). Real-time reactions were performed and analyzed using a Real-Time PCR system (ABI 7300). The relative expression of mRNA was calculated using the 2^−ΔΔCT^ method with GAPDH as the endogenous control. The primer sequences were as follows: TINCR, 5′-GGACAACCTTAGCGTGTTCA-3′ and 5′-TTGGATCAAAGAAGGGAAGG-3′; ANF, 5′-CTC CGATAGATCTGCCCTCTTGAA-3′ and 5′-GGTACCGG AAGCTGTTGCAGCCTA-3′; BNP, 5′-GCTCTTGAAG GACCAAGGCCTCAC-3′ and 5′-GATCCGATCCGGTC TATCTTGTGC-3′; β-MHC, 5′-CAGACATAGAGACCT ACCTTC-3′ and 5′-CAGCATGTCTAGAAGCTCAGG-3′; CaMKII, 5′-GAATCTGCCGTCTCTTGAA-3′ and 5′-TC TCTTGCCACTATGTCTTC-3′ GAPDH, 5′-TGCCCAGA ACATCATCCCT-3′ and 5′-GGTCCTCAGTGTAGCCC AAG-3′.

### Western blotting

The samples were homogenized in 0.1% SDS buffer (Roche, USA) and the lysate was centrifuged at 12,000 rpm for 15 min. The supernatant was collected and the protein concentration was determined using a BCA protein assay kit (Bio-Rad, USA). The extracted protein was separated on SDS-PAGE gel and transferred onto PVDF membrane (Millipore, USA). The membrane was blocked with 5% bovine serum albumin for 1 h to reduce non-specific binding. Then, the blot was incubated with the primary antibody for 12 h at 4°C. The antibodies (CaMKII and β-actin) were purchased from Cell Signaling Technology and used at manufacturer-recommended dilutions (1:1000). After washing, the blot was incubated with HRP-conjugated secondary antibody (Santa Cruz, USA) for 1 h at room temperature. Finally, the signal was detected using the enhanced chemiluminescence kit (Amersham Biosciences) and exposed to X-film.

### Statistical analysis

Data are expressed as mean ± SD, and differences between groups were determined using one-way analysis of variance (ANOVA) with SPSS version 18.0. The Scheffé post-hoc test was used for multiple comparisons if the ANOVA was significant. *P* < 0.05 was considered statistically significant.
